# How many taxa can be recognized within the complex *Tillandsia capillaris* (Bromeliaceae, Tillandsioideae)? Analysis of the available classifications using a multivariate approach

**DOI:** 10.3897/phytokeys.23.4507

**Published:** 2013-05-20

**Authors:** Lucía V. Castello, Leonardo Galetto

**Affiliations:** 1 Instituto Multidisciplinario de Biología Vegetal, CONICET, Universidad Nacional de Córdoba

**Keywords:** *Tillandsia capillaris*, Bromeliaceae, species complex, morphometry, distribution

## Abstract

*Tillandsia capillaris* Ruiz & Pav., which belongs to the subgenus *Diaphoranthema* is distributed in Ecuador, Peru, Bolivia, northern and central Argentina, and Chile, and includes forms that are difficult to circumscribe, thus considered to form a complex. The entities of this complex are predominantly small-sized epiphytes, adapted to xeric environments. The most widely used classification defines 5 forms for this complex based on few morphological reproductive traits: *Tillandsia capillaris* Ruiz & Pav. f. *capillaris*, *Tillandsia capillaris* f. *incana* (Mez) L.B. Sm., *Tillandsia capillaris* f. *cordobensis* (Hieron.) L.B. Sm., *Tillandsia capillaris* f. *hieronymi* (Mez) L.B. Sm. and *Tillandsia capillaris* f. *virescens* (Ruiz & Pav.) L.B. Sm. In this study, 35 floral and vegetative characters were analyzed with a multivariate approach in order to assess and discuss different proposals for classification of the *Tillandsia capillaris* complex, which presents morphotypes that co-occur in central and northern Argentina. To accomplish this, data of quantitative and categorical morphological characters of flowers and leaves were collected from herbarium specimens and field collections and were analyzed with statistical multivariate techniques. The results suggest that the last classification for the complex seems more comprehensive and three taxa were delimited: *Tillandsia capillaris* (=*Tillandsia capillaris* f. *incana-hieronymi*), *Tillandsia virescens* *s. str.* (=*Tillandsia capillaris* f. *cordobensis*) and *Tillandsia virescens* *s. l.* (=*Tillandsia capillaris* f. *virescens*). While *Tillandsia capillaris* and *Tillandsia virescens* *s. str.* co-occur, *Tillandsia virescens* *s. l.* is restricted to altitudes above 2000 m in Argentina. Characters previously used for taxa delimitation showed continuous variation and therefore were not useful. New diagnostic characters are proposed and a key is provided for delimiting these three taxa within the complex.

## Introduction

The subfamily Tillandsioideae comprises 10 genera (Smith and Till 1998, Espejo-Serna 2002, Barfuss et al. 2005) of which *Tillandsia* is the most diversified. In Argentina, *Tillandsia* is represented by 53 species belonging to the subgenera *Anoplophytum* (22 spp.), *Diaphoranthema* (21 spp.), *Phytarrhiza* (7 spp.), and *Allardtia* (3 spp.) (Smith and Downs 1977, Luther and Sieff 1994, Zuloaga et al. 2008). *Tillandsia capillaris* Ruiz & Pav. belongs to the subgenus *Diaphoranthema*, which is characterized by small sized species adapted to arid environments, with abundant absorbing trichomes, inflorescences with few inconspicuous flowers with stamens and styles included in the corolla.

*Tillandsia capillaris* *s. l.* is distributed from southern Ecuador to central Argentina and Chile, between altitudes from 300 m to 4000 m. (Smith and Downs 1977, Till 1989, Jørgensen and León-Yánez 1999). Plants are epiphytes where the canopy is not a limiting factor (Benzing 1990) and colonize different substrates, from native or exotic trees (Astegiano et al. 2007) to exposed rock, and even power lines, walls and metallic fences. Plants produce chasmogamous and cleistogamous flowers (Gilmartin and Brown 1985) and then fruits with a large number of seeds (Till 1992).

In the dry forests of central Argentina (called *Bosque Serrano*, Cabido et al. 2010) *Tillandsia capillaris* *s. l.* is abundant and represents most of the biomass of epiphytes on trees (Astegiano et al. 2007). It has been argued that it causes damage to trees when the abundance is high, due to a decrease in the surface of the host shoot buds (Benzing 1990; Caldiz and Fernández 1995; Soria 2007). Current studies attribute medicinal properties to this taxon (Barboza et al. 2006) and it has been also considered as a bioindicator of air quality (Wannaz et al. 2006).

### The delimitation of the *Tillandsia capillaris* complex and its taxonomic history

The *Tillandsia capillaris* complex constitutes a group of related taxa with a gradual morphological variation. The nomenclatural history itself reflects the complex nature of *Tillandsia capillaris* and allies. The available classifications (Smith and Downs 1977; Till 1989) do not allow an unequivocal recognition of the entities and suggest the existence of gradients between them. These authors considered these plants difficult to identify; Smith (1935, p. 210) mentioned *“this very variable species has a number of forms whose extremes are easily differentiated, but which show all degrees of intergradations in any large collection”*. Otherwise, Till (1989; p. 33) referring to the complex said *“still remains to be clarified by additional studies, if the abundance of the two species have genetic underpinnings, or hybridization processes fade the boundaries between the different forms in both species; names exist in abundance”*.

In the past two centuries several species that are currently included in the *Tillandsia capillaris* complex were described. Ruiz and Pavon (1802) described *Tillandsia capillaris* Ruiz & Pav. and *Tillandsia virescens* Ruiz & Pav. using samples from central Peru. From northern Chile, Gay (1853) described *Tillandsia propinqua* Gay as a related species to *Tillandsia virescens* and *Tillandsia capillaris*. Baker (1878) described *Tillandsia pusilla* Gill. ex Baker, *Tillandsia lanuginosa* Gill. ex Baker and *Tillandsia incana* Gill. ex Baker, emphasizing the similarity of the taxa. Hieronymus (1885) made collection trips in central Argentina, describing *Tillandsia cordobensis* Hieron., *Tillandsia propinqua* Gay var. *saxicola* Hieron., *Tillandsia lichenoides* Hieron. (mistakenly determined by Grisebach in 1874 as *Tillandsia propinqua*) and the variety: *Tillandsia propinqua* var. *saxicola* Hieron. Mez published in 1896 two new species for Argentina: *Tillandsia hieronymi* Mez and *Tillandsia dependens* Hieron. ex Mez, the later with two varieties *Tillandsia dependens* var. *perusneoides* Mez and *Tillandsia dependens* var. *percordobensis* Mez; and named new varieties: *Tillandsia capillaris* var. *incana* Mez, *Tillandsia capillaris* var. *lanuginosa* Mez. Rusby (1910) described *Tillandsia williamsii* Rusby from Bolivia. Finally, Castellanos (1945a) described also for Argentina the species *Tillandsia permutata* A. Cast. and new varieties and forms: *Tillandsia hieronymi* var. *lichenoides* (Hieron.) A. Cast., *Tillandsia virescens* var. *sanzinii* (Hicken) Castell., *Tillandsia dependens* f. *perusneoides* (Mez) Castell., *Tillandsia dependens* f. *percordobensis* (Mez) Castell.

The currently accepted classification in Argentina (Zuloaga et al. 2008) is based on the works of Smith (1935, 1969, 1970) and Smith and Downs (1977), who conducted a review of the genus, defining a single species *Tillandsia capillaris* with 5 forms: *Tillandsia capillaris* Ruiz & Pav. f. *capillaris*, *Tillandsia capillaris* f. *incana* (Mez) L.B. Sm., *Tillandsia capillaris* f. *cordobensis* (Hieron.) L.B. Sm., *Tillandsia capillaris* f. *hieronymi* (Mez) L.B. Sm. and *Tillandsia capillaris* f. *virescens* (Ruiz & Pav.) L.B. Sm. The separation of these forms is based on only four characters: the number of nerves in the floral bracts, the length of the peduncles, the indument of the floral bracts and the size of the leaves.

The lastest revision of the subgenus *Diaphoranthema* in South America was done by Till (1989), who accepted two species, *Tillandsia capillaris* (reducing to the synonymy *Tillandsia capillaris* f. *incana*, and *Tillandsia capillaris* f. *hieronymi*) with a distribution area ranging from southern Ecuador to central Argentina, reaching altitudes of 3500 m; and *Tillandsia virescens* (including *Tillandsia capillaris* f. *virescens* and *Tillandsia capillaris* f. *cordobensis*) with a similar distribution area (the main difference is that it also occurs in Chile), but thriving up to 4300 m. This proposal is also based on a few traits: mainly on differences in the connation of the sepals, the indument of the floral bracts, and the architecture of the sepal veins. Although this classification (Till 1989) can be considered more comprehensive, the classification of Smith and Downs (1977) prevails in the literature.

In this contribution we analyzed the morphological variation of *Tillandsia capillaris* taking as the starting point the five forms defined by Smith and Downs (1977) and cited for Argentina, using 35 floral and vegetative characters with a multivariate approach. These infraspecific taxa are present in many vegetation types in northwestern and central Argentina, where the southern limit of the species is found.

## Materials and methods

Herbarium specimens from CORD and LIL (Holmgren and Holmgren 2001), that were annotated by Lyman B. Smith during his visit to Argentina in 1968 and included in his monograph on Bromeliaceae (Smith and Downs 1977), were used together with new additional specimens from field collections, previously identified with Smith and Downs (1977) keys. The herbarium material inquired by Till during his visit to Argentina in 1990 were also included. Supplementary specimens from B, GOET, MA, P, W, WU were screened but not incorporated in the analyses. A total of 100 specimens were analyzed (20 of *Tillandsia capillaris* f. *capillaris*, 26 of *Tillandsia capillaris* f. *hieronymi*, 19 of *Tillandsia capillaris* f. *incana*, 12 of *Tillandsia capillaris* f. *virescens* and 23 of *Tillandsia capillaris* f. *cordobensis*) from northern and central Argentina, therefore the results are valid for Argentina but not for the whole range of the complex (see supplementary material 1). Each specimen was treated as a taxonomical operational unit (OTU), and 35 floral and vegetative morphological characters were registered, including 12 continuous variables, 7 discontinuous (or discrete) variables, 11 binary variables and 5 multistate variables (Table 1). The morphological features selected include those traits used as key characters in species descriptions by Mez (1896), Castellanos (1945b), Smith and Downs (1977) and Till (1989). All characters were measured in the longest fertile shoot, foliar characters in the most developed leaf, and the character number of leaves per linear cm of shoot in the middle portion of the shoot.

**Table 1.** Qualitative and quantitative characters used for the morphometric study of the complex *Tillandsia capillaris*.<br/>

**Quantitative characters**	**Qualitative characters**
Continuous variables	Binary variables
1-Length of fertile shoot (mm).	5-Type of stem (simple, ramified: 0; 1).
2-Length of stem (mm).	7-Leaf blade apex (rounded, apiculate: 0; 1).
3-Length of leaf blade (mm).	8-Arrangement of the leaf (appressed, non appressed: 0; 1).
4- (half) Width of leaf blade (mm).	10-Leaf sheath exposure (visible, covered by the lower contiguous sheaths: 0; 1).
15-Length of scape (mm).	12-Type of peltate hairs^1^ (type 1+2, type 1+2+3: 0; 1).
18- (half) Width of bract (mm).	16-Scape position (axillary, terminal: 0; 1).
19-Length of floral bract (mm).	20-Floral bract shape (round and wide, triangular and elongated: 0; 1).
25-Length of the sepals (mm).	21-Floral bract apex (acute, mucronate: 0; 1).
30-Length of fruit (mm).	26-Sepal dimension (exceeding the bract, equaling the bract: 0; 1).
31- (half) Width of fruit (mm).	29-Sepal shape: (ovate-lanceolate, acute: 0; 1)
34-Length of seed (mm).	32-Endocarp shape (shaped, not shaped: 0; 1).
35-Length of embryo (mm).	
Discontinuous (or discrete) variables	Multistate variables
6-Number of branches (n°).	9-Type of leaf blade (straight, half-curved, curved: 0; 1; 2).
11-Number of leaves per linear cm of shoot (nº).	13-Winged trichomes position in the leaf (only in the base, in the base and in the middle part, in the whole leaf: 0; 1; 2).
17-Number of inflorescences per branch (n°).	14-Pilosity of the leaf (low, medium, high: 0; 1; 2).
22-Number of nerves in the floral bract (n°).	24-Floral bract indument (glabrous, half pubescent, pubescent: 0; 1; 2).
23-Number of nerves joined together at the apex of bracts (n°).	33-Exocarp shape (straight, curved, very curved: 0; 1; 2).
27-Association degree of the adaxial sepals (%).	
28-Association degree of the abaxial sepals (%).	

^1^Types of trichomes: 1-radially symmetric, 2-one developed wing, 3-two wings developed

### Statistical analyses

Non-parametric Kruskal-Wallis tests (KW) were run for all the variables among the taxa considered. Box-plots were made for continuous variables.

A two-steps analysis was carried out to detect the most informative characters. First, a Principal Component Analysis (PCA) was run using all characters (Woods et al. 2005, Denham et al. 2006, Blanco-Dios 2007, Nicolalde-Morejón 2005), obtaining a correlation matrix with the Pearson coefficient (Sokal and Rohlf 1995), and selecting afterwards characters with coefficient >0.20 as input for a second analysis. With the new matrix (which contained less variables), a Principal Coordinates Analysis (PCoA) was run, using the Gower coefficient (Gower 1971; Hernández 1997; Correa et al. 2007). Dispersion graphs were done for PCA and PCoA with INFOSTAT software (Di Rienzo et al. 2009).

## Results

**Figure 1.**
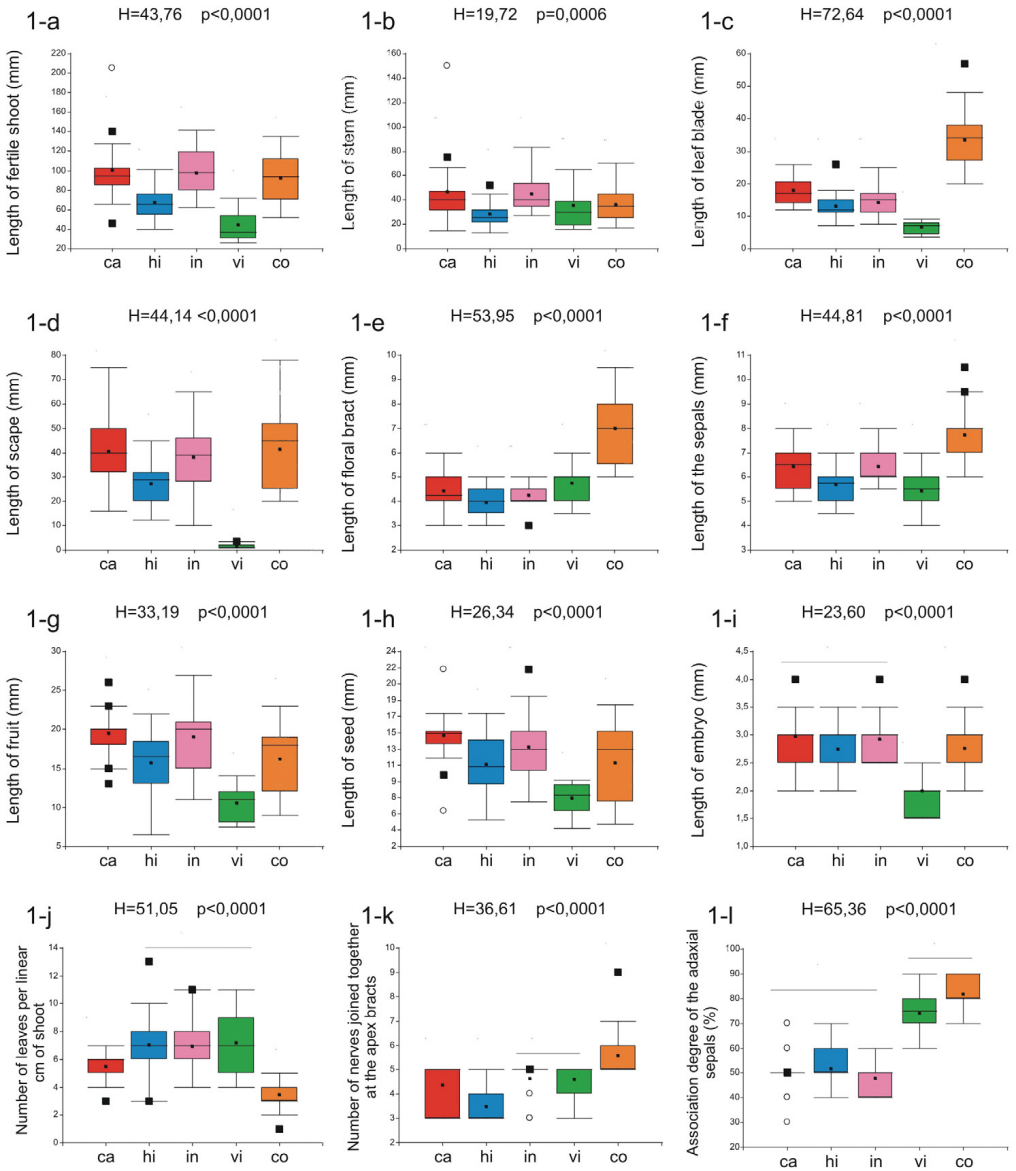
Quantitative analyses of reproductive and vegetative traits in the complex *Tillandsia capillaris* in Argentina. Box plots featuring medians (solid black square), means, and first and third quartiles (large box). Kruskal-Wallis (H) tests performed of selected characters are also included. Different letters above box-plots indicate statistical differences among taxa using a posteriori Dunn tests (p=0,05) (Balzarini et al. 2008). References: OTUs: ca: *capillaris* (n=21); hi: *hieronymi* (n=24); in: *incana* (n=20); vi: *virescens* (n=12); co: *cordobensis* (n=23).

Results of character comparisons showed significant differences (Fig. 1; except for “number of branches” – KW test; *H*=4.68; *P*= 0.24) among the putative taxa but with unclear trends (Fig. 1). For example, *Tillandsia capillaris* f. *virescens* showed significantly lower values compared to the other taxa for several traits (length of: fertile shoot, leaf blade, scape, fruit, seed and of embryo; Figs 1a, c, d, g, h, i respectively). *Tillandsia capillaris* f. *cordobensis* differed from all the other forms by the longer size of leaves, of floral bracts, of sepals, the lower number of leaves per linear cm of shoot and the highest number of nerves joined together at the apex of bracts (Figs 1c, e, f, j, k, respectively). *Tillandsia capillaris* f. *hieronymi* showed significant differences with an intermediate size of the fertile shoot, the scapes, and lower number of nerves joined together at the apex of bracts (Figs 1a, d, k). *Tillandsia capillaris* f. *capillaris* and *Tillandsia capillaris* f. *incana* did not show significant differences and these forms are overlapped with the other forms considering this set of characters (Figs 1a–l).

**Figure 2.**
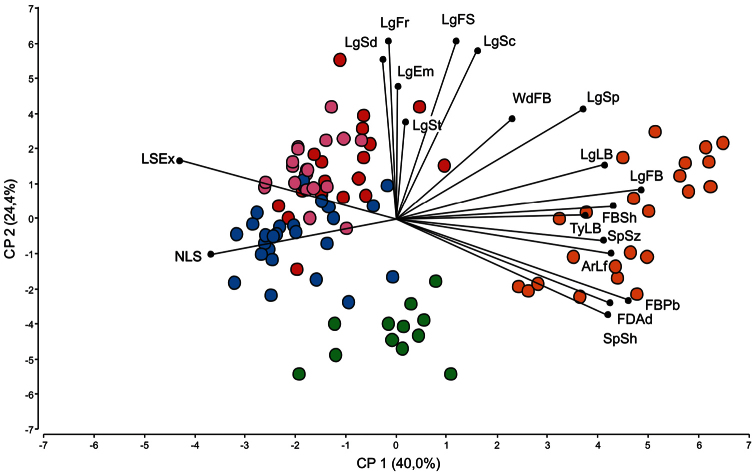
PCA for 5 different taxa of the *Tillandsia capillaris* complex. Plot of all specimens (100 OTUs) and leaning of the most influential 19 characters represented on the first two principal components resulting from principal component analysis (see Table 2 for abbreviations). References: OTUs: f. *capillaris* (n=21) =red; f. *hieronymi* (n=24) =blue; f. *incana* (n=20) =pink; f. *virescens* (n=12) =green; f. *cordobensis* (n=23) =orange.

In the PCA, the first three components explained 50.5 % of variability (25.9, 16.5, and 8.1 % respectively) (results not shown). Analyzing the variables individually, only 19 variables were selected to explain the variance among taxa (see material and methods), considering the ones which showed values up to 0.20 (Table 2). A second PCA using these 19 characters showed that the principal two axes provide a clear ordination of the OTUs into separate groups (Figure 2). The two principal axes together account for 64.4% of the variability. The variance of the first component included quantitative variables (lengths of leaf blade, bract, and sepals, number of leaves per linear cm of shoot, and fusion degree of the adaxial sepals), and qualitative variables (arrangement of the leaf, sepal dimension, type of leaf blade, floral bract shape, sepal shape, indument of the floral bract and leaf sheath exposure). The variance of the second component was supported by quantitative variables concerning the vegetative and the inflorescence size (lengths of fertile shoot, stem, scape, fruit, seed, embryo, and width of bract; Table 2). Figure 2 shows the grouping tendency among the OTUs for this set of 19 variables.

**Table 2.** Title: Principal components analysis results for the *Tillandsia capillaris* complex. Legend: PCA results for 5 taxa of the complex *Tillandsia capillaris* using 35 quantitative and qualitative traits (see M&M for details). The percentages of variance for the two principal components were obtained in the PCA analysis from all the characters. * indicates the values >0.20<br/>

	**Principal components**	
Character	Axis 1	Axis 2
Length of fertile shoot (LgFS)	0.06	0.36*
Length of stem (LgSt)	3.8E-03	0.22*
Length of leaf blade (LgLB)	0.25*	0.12
Width of leaf blade (WdLB)	-0.08	0.19
Length of scape (LgSc)	0.08	0.33*
Width of floral bract (WdFB)	0.14	0.23*
Length of floral bract (LgFB)	0.29*	0.08
Length of the sepals (LgSp)	0.22*	0.24*
Length of fruit (LgFr)	-0.03	0.34*
Width of fruit (WdFr)	0.12	0.19
Length of seed (LgSd)	-0.03	0.29*
Length of embryo (LgEm)	-0.01	0.25*
Number of branches (NBr)	-0.03	0.09
Number of leaves per linear cm of shoot (NLS)	-0.22*	-0.08
Number of inflorescences per branch (NIB)	-0.04	0.09
Number of nerves in the floral bract (NNB)	0.15	0.17
Number of nerves joined together at the apex of bracts (NNA)	0.20	0.11
Fusion degree of the adaxial sepals (FDAd)	0.27*	-0.14
Fusion degree of the abaxial sepals (FDAb)	-0.11	0.14
Type of stem (TySt)	-0.04	0.08
Leaf blade apex (LBAp)	-0.13	-0.02
Arrangement of the leaf (ArLf)	0.26*	-0.05
Type of leaf blade (TyLB)	0.22*	-4.9E-03
Leaf sheath exposure (LSEx)	-0.27*	0.10
Type of peltate hairs (TyPH)	0.01	0.09
Winged hairs position in the leaf (WHPL)	-0.16	0.20
Pilosity of the leaf (PiLf)	-0.16	0.05
Scape position (ScP)	0.06	-0.13
Floral bract shape (FBSh)	0.26*	0.04
Floral bract apex (FBA)	0.05	-0.05
Floral bract indument (FBPb)	0.29*	-0.14
Sepal dimension (SpSz)	0.25*	-0.03
Sepal shape (SpSh)	0.27*	-0.16
Endocarp shape (EnSh)	0.05	-0.06
Exocarp shape (ExSh)	-0.09	0.07

**Figure 3.**
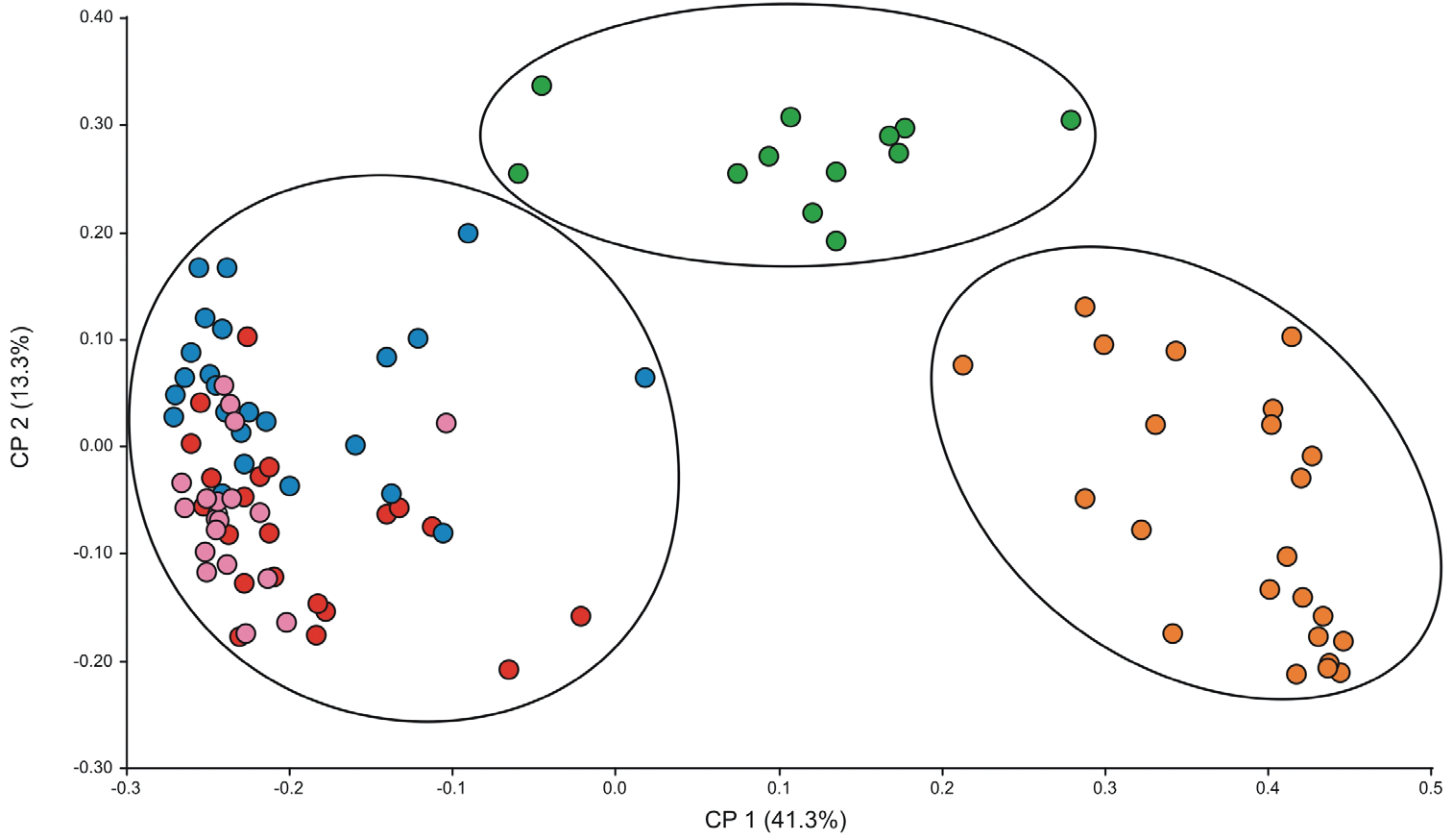
Principal coordinates analysis (PCoA) for 5 different taxa of the *Tillandsia capillaris* complex. Scatterplots of the first two axis based on 19 characters selected in the PCA and using the Gower distance (sqrt (1-S)). References: Characters used (see Table I); OTUs: f. *capillaris* (n=21) =red; f. *hieronymi* (n=24) =blue; f. *incana* (n=20) =pink; f. *virescens* (n=12) =green; f. *cordobensis* (n=23) =orange.

PCoA showed that the two principal axes provide a clear ordination of the OTUs into three separate groups (Fig. 3). The two principal axes together account for 54.6% of the variability using the 19 characters previously selected in the PCA with coefficient >0.20 (Fig. 3). There is a clear distinction with a larger left-group formed by the *Tillandsia capillaris* f. *capillaris*, *Tillandsia capillaris* f. *hieronymi* and *Tillandsia capillaris* f. *incana* OTUs; a second central, upper-group formed by the *Tillandsia capillaris* f. *virescens* OTUs; and a third lower, right-group corresponding to the OTUs for *Tillandsia capillaris* f. *cordobensis*.

**Figure 4.**
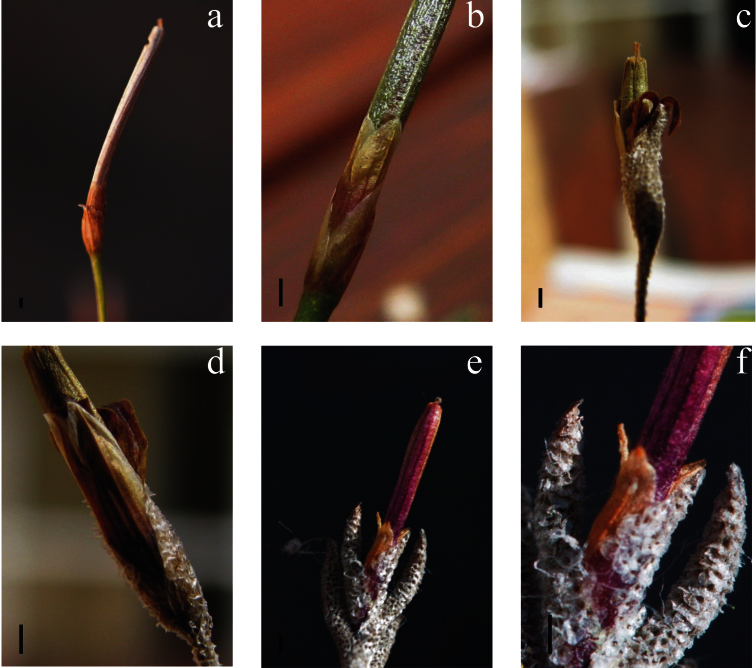
Infructescence structure in *Tillandsia capillaris* complex. **a–b** *Tillandsia capillaris* (=*Tillandsia capillaris* f. *incana* and *Tillandsia capillaris* f. *hieronymi*) **a** glabrous floral bracts much shorter than the sepals **b** the ovate-lanceolate sepals are partially fused **c–d** *Tillandsia virescens* s.str. (=*Tillandsia capillaris* f. *cordobensis*) **c** pubescent floral bracts equaling the sepals **d** the acute sepals are much more fused (60-90%) **e–f** *Tillandsia virescens* *s. l.* (=*Tillandsia capillaris* f. *virescens*) **e** pubescent floral bracts equaling the sepals, lacking scapes and violet capsules **f** the acute sepals are almost totally fused. Abbreviations: *s*=sepals; *b*=floral bract, bars=1 mm.

Many of the 19 most influential characters are useful to separate *Tillandsia capillaris* f. *incana-hieronymi* (=*Tillandsia capillaris* sensu Till) (Fig. 4a, b), *Tillandsia capillaris* f. *cordobensis* (=*Tillandsia virescens* *s. str.* sensu Till) (Fig. 4c, d) and *Tillandsia capillaris* f. *virescens* (=*Tillandsia virescens* *s. l.* sensu Till) (Fig. 4e, f). For example, characters such as: triangular and elongated floral bract; sepals long, acute and equaling the bract; elongated and curved leaf blades; and low number of leaves per linear cm of shoot are useful to delimit *Tillandsia capillaris* f. *cordobensis*.The second group formed by *Tillandsia capillaris* f. *capillaris*, *Tillandsia capillaris* f. *incana* and *Tillandsia capillaris* f. *hieronymi* can be circumscribed by: ovate-lanceolate sepals, exceeding in length the floral bract; round and wide floral bract; and straight and half-curved leaf blade. Finally, *Tillandsia capillaris* f. *virescens* (=*Tillandsia virescens* *s. l.*) has the smaller sizes of the fertile shoot, scape, leaf blade, fruit, seed, and embryo. This last form showed statistically similarities in some of the characters (length of the fertile shoot, scape and sepals; Figs 1a, d, f, respectively) with *Tillandsia capillaris* f. *hieronymi*. Nevertheless, the characters indument of the bract, shape of the sepals and fusion degree of the adaxial sepals allowed to separate the forms in two different groups (Fig. 2).

### Key for the recognition of the morphotypes proposed for the *Tillandsia capillaris* complex in Argentina

**Table:** 

1	Floral bracts glabrous. Sepals ovate-lanceolate, partially fused, exceeding in length the floral bracts	*Tillandsia capillaris* (=*Tillandsia capillaris* f. *incana-hieronymi*)
–	Floral bracts pubescent or semi-pubescent. Sepals acute, almost totally fused, equaling or barely exceeding in length the floral bracts.
2	Scapes developed (2–8 cm in length). Leaf blades elongated and curved, 2–6 cm long. Low number of leaves per linear cm of shoot (<4–5 leaves). Leaf sheaths widely visible. Sepals acute connated by 60–90% of their lengths, with 5–9 nerves. Floral bracts triangular and elongate	*Tillandsia virescens* *s. str.* (=*Tillandsia capillaris* f. *cordobensis*)
–	Scape absent or scarcely developed (1-3.5 mm in length). Leaf blades straight and half-curved, shorter than 1 cm. Leaves per linear cm of shoot 5–11. Leaf sheaths barely visible. Sepals acute almost totally fused and with 1–3 nerves. Floral bracts round and wide	*Tillandsia virescens* *s. l.* (=*Tillandsia capillaris* f. *virescens*)

## Discussion

The criteria used in previous classifications (Smith and Downs 1977, Till 1989) are not satisfying to resolve the complexity of this group. Nevertheless, when the number of qualitative and quantitative characters is increased, a pattern emerged through a multivariate test allowing the separation of the putative taxa into three recognizable groups. The application of morphometric studies in the Bromeliaceae currently prevails in the literature to resolve different complex groups (Wendt et al. 2000, Costa et al. 2009, Pinzón et al. 2011) stressing the potential of this statistical tool to evaluate the limits between closely related taxa (Sokal and Rohlf 1995).

Among the characters analyzed, most were informative (approximately 63% of the quantitative and 44% of the qualitative characters). The quantitative characters are significant to separate groups, while the categorical characters were useful when the taxa had similar plant size (e.g. *Tillandsia capillaris* f. *virescens* and *Tillandsia capillaris* f. *hieronymi*). Within the non-informative qualitative characters, those referred to the peltate trichomes (TyPH, WHPL and PiLf) were cited in previous classifications (Hieronymus 1885). We suggest to avoid the use of these characters for taxonomic purposes, unless a detailed morphometric study is done. Once, environmental variation for the number of trichomes and the size of the wing area in *Tillandsia* has been suggested (Stefano et al. 2008).

Diagnostic characters used by Smith and Downs (1977) were useless to differentiate the forms *Tillandsia capillaris* f. *capillaris*, *Tillandsia capillaris* f. *hieronymi* and *Tillandsia capillaris* f. *incana* ocurring in Argentina, because these taxa showed a gradual variation in many characters (e.g., the number of nerves in the floral bract, the length and position of the scape, the length and diameter of the leaves).For example, *Tillandsia capillaris* f. *incana* described by Smith and Downs (1977) with short, wide and appressed leaf blades did not show statistically significant differences in any of these characters when it was compared with the other forms. *Tillandsia capillaris* f. *hieronymi* was previously circumscribed by the 3 nerves joined together at the apex of the floral bract (Smith and Downs 1977). Nevertheless, this was a variable character, varying from 3-5 nerves in the three forms of the “*capillaris”* complex defined here. Also, *Tillandsia capillaris* f. *capillaris* did not show statistical differences in the scape position (Smith and Downs 1977). This variable seems to be related with the development of the stem (Castellanos 1945b), and it was registered for all the forms terminal and axillary inflorescences.

On the contrary, other diagnostic characters established by Smith and Downs (1977) to determine *Tillandsia capillaris* f. *cordobensis* and *Tillandsia capillaris* f. *virescens* showed statistically significant differences. *Tillandsia capillaris* f. *cordobensis* can be circumscribed by both the indument of the floral bracts (Fig. 4c, d) and wide spreading leaves (the leaf sheaths are prominent and visible so that the leaf blades are detached). In addition, some complementary characters allow an easier delimitation of this taxon, as the exposure of the leaf sheaths or the leaf density per cm of stem. *Tillandsia capillaris* f. *virescens* can be recognized by the lacking of a scape (Smith and Downs 1977) (Fig. 4e, f). It is interesting to note that the reduction of the reproductive organs in this taxon could be a feature related to environment constraints (altitude) where this form lives (Gilmartin and Brown 1985).

Till (1989) used the fusion degree of the adaxial and abaxial sepals as the main character to delimit the complex into two groups: one with adaxial sepals partially fused (20-60%) (Fig. 4b) and abaxial sepals fused by their bases (10-35%) (*Tillandsia capillaris*=*Tillandsia capillaris* f. *incana-hieronymi*); a second group with adaxial sepals more fused (50-90%) (Fig. 4d, f), and abaxial sepals less fused (5-20%) (*Tillandsia virescens* *s. str.* and *s. l.*=*Tillandsia capillaris* f. *cordobensis* and *Tillandsia capillaris* f. *virescens*). Data for the material studied here showed values of 40-70% fusion for the adaxial sepals in the first group (*Tillandsia capillaris*=*Tillandsia capillaris* f. *incana- hieronymi*), and 60-90% in the second group (*Tillandsia virescens* *s. str.* and *s. l.*=*Tillandsia capillaris* f. *cordobensis* and *Tillandsia capillaris* f. *virescens*). Although the trend described by Till (1989) for the abaxial sepals was detected, statistical differences supported the separation of the complex into three groups. Till (1984) considered *Tillandsia virescens* as one species, and *Tillandsia cordobensis* as a taxonomic synonym of *Tillandsia virescens* (Till 1984, p. 135-136), and defined five aggregates for “*Tillandsia virescens*”. After revising the herbarium material that he studied, we interpreted that *Tillandsia virescens* *s. str.* (=*Tillandsia capillaris* f. *cordobensis*) comprise “group 1: *Tillandsia cordobensis*” and “group 2: *Tillandsia cordobensis* “var.” *tucumanensis* nom. nud.”. Otherwise, *Tillandsia virescens* *s. l.* (=*Tillandsia capillaris* f. *virescens*) include “group 4: *Tillandsia propinqua* “var.”” (Till 1984). Till (1989) also used the architecture of the veins of the sepals as a character, but we did not consider it here because to exam such feature we would cause severe damage to herbarium specimens. Other characters briefly mentioned by Till (1989; shape of the bract and size of the sepals) were measured here and were significant to separate the taxa.

Summarizing, our results partially support the classification of Till (1989) that considered the first group as *Tillandsia capillaris* since the OTUs for *Tillandsia capillaris* f. *capillaris*, *Tillandsia capillaris* f. *hieronymi* and *Tillandsia capillaris* f. *incana* tend to form a single ensemble. On the other hand, concerning the differences between *Tillandsia capillaris* f. *cordobensis* and *Tillandsia capillaris* f. *virescens*, the classification by Smith and Downs (1977) is still appropiatted. However, we are also evaluating the taxonomical thesis of Till (1984) and considered his classification (“*Tillandsia virescens* and aggregates”) in future taxonomical work for the complex, since we saw that the characters he used (the cohesion of the sepals and the indument of the floral bract) were useful to defined these groups. We expect to propose conclusive nomenclatural changes, however, only after gathering additional data. Taxonomic resolution of complex groups, ideally, should be done combining morphological data from the whole range of distribution.

### The *Tillandsia capillaris* complex in Central Argentina

All the forms analyzed are distributed in the central and northern Argentina, in the southern distributional range of the complex. The taxa *Tillandsia capillaris* (=f. *incana- hieronymi*) and *Tillandsia virescens* *s. str.* (=f. *cordobensis*), co-occur in almost the same sites and altitude levels. *Tillandsia virescens* *s. str.* was mentioned by Smith and Downs (1977) for altitudes above 900 m. We found populations at lower altitudes (300 m), co-occurring with *Tillandsia capillaris*. On the other hand, *Tillandsia virescens* *s. l.* (=f. *virescens*), with saxicolous habit, was found restricted to higher altitudes in the central Argentina (above 2000 m; Achala batholith), or in the western foothill of the Andes (between 2000-3500 m). It is interesting to note that in some regions and at higher altitudes (2000 m, for example in the central Argentina), the three forms can co-occur, but at lower altitudes (as in the woodland mountains of Bosque Serrano at, 400-1100 m) only two of these forms can be found (*Tillandsia capillaris* and *Tillandsia virescens* *s. str.*).

## Conclusion

The main goal of this contribution was to analyze the available classifications of the *Tillandsia capillaris* complex using a relatively large sample of material with a multivariate perspective. This methodological approach allowed us to define three taxa in Argentina (Figs 3 and 4) with clear morphological limits, and to inquire into the conflicts between the available classifications. The next step is to compare these results using new material from other populations within the geographical range of the complex, specifically from Bolivia and Peru. We are not proposing new nomenclature combinations until the whole distribution area are investigated.

## Acknowledgements

We thank Jorge Chiapella, Walter Till, and Sabina Donadío for useful taxonomic discussions on the complex. Also to Leonardo Versieux, Andrea Costa, Walter Till and Jorge Chiapella for helpful comments and suggestions of previous version of this manuscript. We are indebted to the curators of CORD, LIL, MA, W, and WU for access to plant material. We thank also to Marcelo Gritti that took some of the photographs. Financial support was provided by CONICET, SECyT (UNC), MINCyT and BMFW.
